# A hyperadrenergic state in hypertension is associated with depressive symptoms and impaired stress-modulated vasomotor responses: evidence for chronic stress as a common aetiology for a hypertension/depression phenotype

**DOI:** 10.1192/bjo.2025.64

**Published:** 2025-08-11

**Authors:** Bushra Farukh, Luca Faconti, Ryan J. McNally, Calum D. Moulton, Allan H. Young, Phil J. Chowienczyk

**Affiliations:** British Heart Foundation Centre, School of Cardiovascular and Metabolic Medicine & Sciences, King’s College London, UK; Department of Psychological Medicine, Institute of Psychiatry, Psychology & Neuroscience, King’s College London, UK

**Keywords:** Hypertension, depression, anxiety, chronic stress, noradrenergic activity

## Abstract

**Background:**

Noradrenergic activation in the central and peripheral nervous systems is a putative mechanism explaining the link between hypertension and affective disorders.

**Aims:**

We investigated whether these stress-sensitive comorbidities may be dependent on basal noradrenergic activity and whether vascular responses to centrally acting stimuli vary according to noradrenergic activity.

**Method:**

We examined the relation of affective disorders and stress-mediated vascular responses to plasma concentrations of normetanephrine, a measure of noradrenergic activity, in subjects with primary hypertension (*n* = 100, mean ± s.d. age 43 ± 11 years, 54% male). The questionnaires Patient Health Questionnaire-9 (PHQ-9), 16-item Quick Inventory of Depressive Symptomatology-Self Report (QIDSSR-16) and Generalized Anxiety Disorder-7 (GAD-7) were used for evaluation of symptoms of depression and anxiety. Forearm blood flow (strain gauge plethysmography) was used to assess vascular responses to mental stress and to device-guided breathing (DGB), interventions that respectively increase or decrease noradrenergic activity in the prefrontal cortex and locus coeruleus.

**Results:**

Low mood and high anxiety were two- to threefold higher for hypertensive subjects in the highest compared with the lowest normetanephrine tertiles (each *P* < 0.005). Forearm vasodilator responses to mental stress and vasoconstrictor responses to DGB were attenuated in those with high compared with low normetanephrine (28.3 ± 21% *v*. 47.1 ± 30% increases for mental stress and 3.7 ± 21% *v*. 18.6 ± 15% decreases for DGB for highest versus lowest tertiles of normetanephrine, each *P* ≤ 0.01).

**Conclusions:**

A hyperadrenergic state in hypertension is associated with mood disturbance and impaired stress-modulated vasomotor responses. This association may be mediated by chronic stress impinging on pathways regulating central arousal and peripheral sympathetic nerve activity.

Affective disorders and hypertension are among the most common chronic mental and physical conditions that account for a large proportion of premature death and disability worldwide. The two conditions cluster, occurring together more commonly than would be expected from their individual prevalence rates.^1–4^ Individuals with hypertension are estimated to be 1.76 times more likely to have moderate to severe symptoms of depression (Patient Health Questionnaire, PHQ-9 > 10) compared with those without hypertension (odds ratio 1.76, 95% CI: 1.14–2.74).^5^ Furthermore, both hypertension and depression are risk factors for cardiovascular disease and mortality.^6,7^ However, there is poor understanding of the biological mechanisms underpinning the association between hypertension and depression.

Chronic stress, a factor commonly linked with the development of depression and hypertension, causes tonic activation of the locus coeruleus, which enhances sympathetic tone and increases norepinephrine turnover. This effect is particularly evident in the prefrontal cortex and hippocampus, and is mediated by the amplification of sympathetic tone in the peripheral nervous system.^8–11^ Consequently, pharmacological interventions targeting the sympathetic nervous system could improve depression and hypertension concurrently while reducing the associated risk of cardiovascular disease. However, beyond a small number of studies examining urinary catecholamines,^12,13^ the mechanistic link between depression and hypertension has not been explored.

In the present study we examined the association of symptoms of affective disorders with plasma concentrations of normetanephrine, a measure of chronic sympathetic nervous system activation,^14^ in patients with primary hypertension. We also examined responses to mental stress and to device-guided breathing (DGB), interventions that act through the same central pathways implicated in chronic stress, to stimulate or inhibit stress-induced vascular responses, and examined the association of these responses with plasma concentrations of normetanephrine. We hypothesised that chronic sympathetic nervous system activation, as indicated by high plasma normetanephrine concentrations, would be associated with worse anxiety and depressive symptoms, and with impaired stress-modulated vasomotor responses.

## Method

### Study design and population

Subjects with primary hypertension and aged ≥18 years were consecutively recruited from the hypertension clinic at Guy’s and St Thomas’ Hospital NHS Foundation Trust, London, UK. Hypertension was diagnosed based on previous anti-hypertensive treatment and/or daytime ambulatory blood pressure ≥135 mmHg systolic and/or 85 mmHg diastolic, in line with current guidelines from the European Society of Hypertension.^15^ Exclusion criteria included pregnancy, subjects with secondary hypertension (including hypertension secondary to drug use), use of antidepressants or beta-blockers, renal impairment, established cardiovascular diseases other than hypertension or any other significant comorbidities apart from hypertension mood disturbance. The authors assert that all procedures contributing to this work comply with the ethical standards of the relevant national and institutional committees on human experimentation, and with the Helsinki Declaration of 1975 as revised in 2013. All procedures were approved by the local Research Ethics Committee (ref. no. 12/LO/1473). Written informed consent was obtained from all participants.

### Demographics and anthropometrics

Detailed social and medical history, including smoking status, alcohol consumption, treatment and comorbidities, was obtained followed by blood sampling for biochemistry, anthropometric measurements and cardiovascular stress responses. Diabetes was diagnosed based on either previous/current diabetes treatment or glycosylated haemoglobin (HbA1c) ≥6.5% (48 mmol/mol) in untreated individuals. Ethnicity was assigned as ‘self-defined ethnicity’, grouped into either White or Black and minority ethnicity.

### Depression, anxiety and sleep disturbance

Self-report questionnaires in written format were used to assess depression, anxiety and sleep symptom severity. The Patient Health Questionnaire (PHQ-9) and the 16-item Quick Inventory of Depressive Symptomatology- Self Report (QIDSSR-16) were used to determine depressive symptoms; Generalized Anxiety Disorder-7 (GAD-7) for anxiety; and Epworth Sleepiness Score (ESS) and STOP-BANG for evaluation of sleep disturbance and presence of obstructive sleep apnoea (OSA). For these questionnaires, higher scores indicate worse symptom severity. These questionnaires are psychometrically robust and commonly used in adult populations, exhibiting high test–retest reliability and high concurrent validity.^16–20^

### Biochemistry analysis

Blood biochemistry analysis was performed at ViaPath Laboratories, Guy’s and St Thomas’ Trust, London, UK. Plasma biochemistry was determined from samples obtained following 15 min of rest in supine position. Blood samples were collected from the antecubital fossa into ethylenediaminetetra-acetic acid-containing tubes, mixed, placed on ice then centrifuged within 30 min of collection. Plasma was aliquoted into cryovials and stored at −80°C. Assays were performed in duplicate from these aliquots with internal quality controls, maintaining inter- and intra-assay coefficients of variation below 10%. Measurements included creatinine, electrolytes, fasting glucose, lipid profile, plasma renin, aldosterone, normetanephrine and metanephrine. Normetanephrine is a metabolite of norepinephrine that, in the absence of a catecholamine-secreting tumour, derives predominantly from peripheral sympathetic nerves. From the perspective of the present application, it has the advantages over norepinephrine in that it is less influenced by acute stress and that plasma concentrations of normetanephrine taken under basal conditions hence represent chronic activity of the sympathetic nervous system.^14^

### Stress-induced cardiovascular responses

Participants were asked to abstain from caffeine, strenuous exercise and alcohol for 24 h before the visit. All measurements were performed in a quiet, temperature-controlled vascular laboratory (23–25°C). Cardiovascular measurements were performed at baseline following ≥15 min of rest in supine position, then during mental stress and DGB with these interventions performed consecutively in random order and with the second intervention following a period of rest, after which further baseline measurements were recorded. Mental stress was elicited using the Stroop colour-word test for a period of 5 min. This is a standardised test utilised as a cognitive stressor capable of inducing emotional responses and heightened levels of autonomic reactivity.^21,22^ DGB was performed using the Resperate device (InterCure Ltd., Lod, Israel). This is a biofeedback device consisting of a respiration sensor and headphones with feedback sounds generated to prolong the expiratory phase of each breath, thereby reducing the breathing rate to <10/min.

Cardiovascular measurements comprised blood pressure, heart rate and forearm blood flow (FBF). Brachial blood pressure was obtained using an Omron HEM 705-CP semiautomatic oscillometric recorder (Omron Health Care, Tokyo, Japan). An average of three consecutive readings of systolic blood pressure (SBP), diastolic blood pressure (DBP) and heart rate was recorded. FBF was measured using venous occlusion strain gauge plethysmography (Hokanson, Inc., Washington, USA) with strain gauges that were electrically calibrated. Cuffs placed on the wrist were inflated to a suprasystolic blood pressure level to prevent any contribution of hand blood flow, whereas venous collecting cuffs on the biceps were inflated intermittently to 40 mmHg for 10 s. FBF was measured from the initial increase in arm circumference for an average of five or more upper arm cuff inflations. Forearm vascular resistance (FVR) was calculated from mean arterial pressure (MAP) divided by FBF.

### Statistical analysis

Descriptive summary statistics were stratified according to tertiles of plasma normetanephrine determined following the recruitment of the cohort. Mean ± s.d. was used for continuous variables (unless otherwise stated), and counts and percentages for categorical variables. Vasodilation/vasoconstriction and FVR were analysed as absolute numerical change, as well as by percentage change from immediately preceding baseline measurements. Differences between groups were analysed using one-way analysis of variance (ANOVA) and analysis of covariance (ANCOVA) for normally distributed values. Post hoc Bonferroni analysis was used to assess the significance of differences between groups. The independent-samples Kruskal–Wallis test was utilised for non-normally distributed variables, with ANCOVA used to adjust for confounding factors following log-transformation of non-normally distributed variables. A *χ* ^2^ test was used for categorical values.

All tests were two-tailed, and differences were considered significant with at *P* < 0.05. Using G*Power 3.1, we calculated that a sample size of 100 patients would be sufficient to detect moderate to large differences (Cohen’s *f* = 0.32) across groups at 80% power and *P* < 0.05.^23^

SPSS Statistics (version 25 for Windows) was used for all statistical analyses, and GraphPad Prism 9 (for Windows; GraphPad Software Inc., La Jolla, California, USA; https://graphpad.com) for all graphical representation of data.

## Results

The characteristics of hypertensive subjects (*n* = 100, from a total of 106 approached and 6 declining due to time constraints), stratified according to tertiles of normetanephrine, are shown in Table 1. The study population had a mean age of 43.4 ± 10.9 years, with 54% of participants male; 60% of participants were from Black and minority ethnic groups, in line with the demographic of South-east London where the hospital is located. The majority of subjects (88%) were being treated with anti-hypertensive medications, and 4% had type 2 diabetes. Age, gender and ethnicity were similar across the tertiles of normetanephrine, as was the proportion of smokers and those regularly consuming alcohol. However, body mass index (BMI) was greater in those in the highest compared with the lowest tertile of normetanephrine distribution (30.8 ± 5.0 *v*. 27.1 ± 4.0 kg/m^2^, *P* = 0.012).

**Table 1:** Demographics, questionnaire scores and biochemistry analysis stratified by tertiles of plasma normetanephrine (NM)

Parameters	Plasma NM tertiles		
	1 (low NM), *n* = 36	2 (mid NM), *n* = 32	3 (high NM), *n* = 32
Plasma NM, pmol/L	352.83 ± 75.27	666.38 ± 163.24	1151.22 ± 242.47**
Demographics and anthropometrics			
Age, years	40.97 ± 11.29	44.78 ± 9.81	44.59 ± 11.25
Male, %	55.5	50.0	56.3
BMI, kg/m^2^	27.11 ± 4.02	30.06 ± 6.42	30.79 ± 4.97*
Alcohol consumption, %	69.4	66.7	68.3
Active smokers, %	12	7	14
Anti-hypertensive treatment, %			
Diuretics	37.2	30.4	33.3
ACE inhibitors	33.3	40.6	43.8
Angiotensin	8.3	12.5	12.5
Alpha-blockers	5.6	3.1	9.4
Ca channel blockers	47.2	34.4	55.6
Questionnaire responses			
PHQ-9	3.76 ± 2.67	5.79 ± 5.10	9.16 ± 5.80**
QIDSSR-16	4.48 ± 3.48	6.76 ± 5.16	12.26 ± 4.91*
GAD-7	4.58 ± 3.84	5.70 ± 4.82	8.96 ± 5.45*
Epworth Sleepiness Score	6.26 ± 3.24	6.02 ± 3.75	9.27 ± 4.58**
STOP-BANG	2.73 ± 1.28	3.32 ± 1.42	4.48 ± 2.04**
Biochemistry			
Creatinine, µmol/L	84.68 ± 24.90	80.69 ± 15.19	91.70 ± 25.86
Total cholesterol, mmol/L	4.91 ± 0.89	5.04 ± 0.98	5.10 ± 0.90
HbA1c, mmol/mol	37.25 ± 4.36	39.16 ± 4.40	39.99 ± 5.18*
CRP, mg/L	2.25 ± 1.59	4.79 ± 4.52	5.97 ± 6.84*
Cortisol	249.26 ± 101.29	183.50 ± 81.63	290.45 ± 214.92

BMI, body mass index; ACE, angiotensin-converting enzyme; Ca, calcium; PHQ-9, Patient Health Questionnaire-9; QIDSSR-16, Quick Inventory of Depressive Symptomatology Self Report-16; GAD-7, Generalized Anxiety Disorder-7; HbA1c, glycosylated haemoglobin; CRP, C-reactive protein.

*P < 0.05, **P < 0.001, tertile 3 versus tertile 1.

### Depression, anxiety and sleep questionnaires

**Fig. 1:**
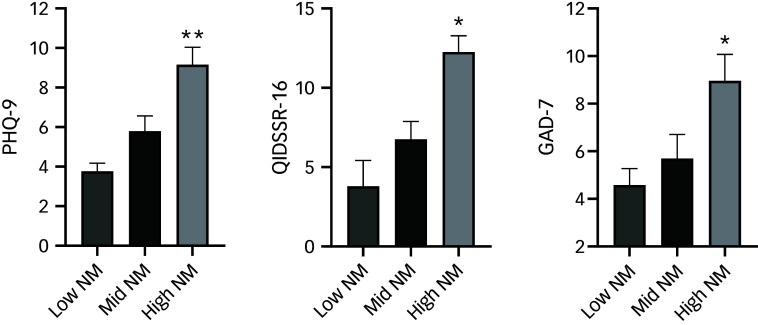
Depression and anxiety questionnaire scores stratified according to tertiles of plasma normetanephrine (NM). PHQ-9, Patient Health Questionnaire-9; QIDSSR-16, Quick Inventory of Depressive Symptomatology Self Report-16; GAD-7, Generalized Anxiety Disorder-7. *P < 0.05, **P < 0.001, tertile 3 (high NM) versus tertile 1 (low NM).

A high proportion of patients had scores for PHQ-9 and QIDSSR-16 indicating significant mood disturbance (31 and 34% of the study population scored moderate to severe depression for PHQ-9 and QIDSSR-16, respectively). Scores representing low mood and high anxiety were two- to threefold higher for hypertensive subjects in the higher compared with the lower tertiles of normetanephrine (Fig. 1). The mean scores for PHQ-9 were 9.16 ± 5.80 in the highest tertile compared with 3.76 ± 2.67 in the lowest (difference in score (95% CI) 5.40 (2.92–7.88), *P* < 0.001), and those for QIDSSR-16 were 12.26 ± 4.91 *v*. 4.48 ± 3.48 (difference 7.78 (95% CI: 4.71–10.84), *P* = 0.003). Similarly, GAD-7 scores were 8.96 ± 5.45 *v*. 4.58 ± 3.84 (difference 4.38 (95% CI: 1.27–7.49), *P* = 0.003) in the highest versus lowest normetanephrine tertiles, respectively. ESS and STOP-BANG scores were also higher in subjects with higher normetanephrine levels (Table 1).

### Blood pressure and stress-induced cardiovascular responses

Baseline blood pressure and FBF were similar across the tertiles of normetanephrine, but heart rate was higher in subjects in the highest compared with the lowest tertile (Table 2). Mental stress resulted in an increase in SBP and DBP that was similar across the tertiles of normetanephrine, but an increase in FBF was attenuated in subjects in the highest compared with the lowest tertile: FBF increased by 47.1 ± 29.7% in the lowest compared with 28.3 ± 21.2% in the highest tertile and FVR decreased by 22.8 ± 12.6% in the low normetanephrine tertile versus 10.5 ± 11.8% in the high tertile (both *P* < 0.01). The increase in heart rate was also lower in subjects in the highest compared with the lowest tertiles (7.8 ± 6.7% *v*. 14.9 ± 10.5%, *P* = 0.01).

**Table 2:** Blood pressure, mental stress and device-guided breathing responses stratified by tertiles of plasma normetanephrine (NM)

Parameters	Plasma NM tertiles		
	Low NM, *n* = 36	Mid NM, *n* = 32	High NM, *n* = 32
Haemodynamics			
SBP, mmHg	141.02 ± 16.57	144.25 ± 19.21	143.25 ± 14.42
DBP, mmHg	88.17 ± 10.70	89.60 ± 12.54	87.86 ± 11.61
MAP, mmHg	105.71 ± 11.42	107.71 ± 13.99	106.22 ± 11.81
HR, bpm	67.38 ± 10.72	70.36 ± 9.01	74.54 ± 12.42*
FBF, 100 mL/mL	3.78 ± 1.28	3.12 ± 1.32	3.54 ± 1.13
FVR, mmHg,100 mL/mL	32.40 ± 15.28	39.55 ± 15.98	32.99 ± 11.04
Mental stress responses (mental stress, baseline)			
ΔSBP, mmHg	12.63 ± 9.29	9.69 ± 7.58	9.68 ± 7.76
ΔDBP, mmHg	10.48 ± 6.12	8.83 ± 5.92	8.84 ± 7.10
ΔMAP, mmHg	11.27 ± 6.89	9.20 ± 5.43	9.26 ± 6.31
ΔHR, bpm	9.79 ± 7.16	6.52 ± 4.46	5.60 ± 4.94*
ΔFBF, 100 mL/mL	1.55 ± 0.61	1.18 ± 0.77	0.93 ± 0.59**
ΔFBF, %	47.14 ± 29.71	43.11 ± 31.39	28.26 ± 21.23*
FVR, mmHg, 100 mL/mL	(−)8.62 ± 9.46	(−)9.07 ± 7.92	(−)3.32 ± 3.79*
FVR, %	(−)22.75 ± 12.56	(−)20.78 ± 16.20	(−)10.48 ± 11.83*
DGB responses (DGB, baseline)	*n* = 35	*n* = 30	*n* = 30
ΔSBP, mmHg	(−)4.42 ± 5.93	(−)5.48 ± 6.35	(−)5.51 ± 8.74
ΔDBP, mmHg	(−)2.11 ± 3.83	(−)2.53 ± 5.58	(−)2.33 ± 5.28
ΔMAP, mmHg	(−)2.65 ± 3.74	(−)3.43 ± 5.27	(−)3.34 ± 5.34
ΔHR, bpm	(−)2.11 ± 3.36	(−)2.87 ± 3.65	(−)4.58 ± 4.88*
ΔFBF, 100 mL/mL	(−)0.68 ± 0.65	(−)0.31 ± 0.81	(−)0.13 ± 0.69*
ΔFBF, %	(−)18.55 ± 15.22	(−)8.84 ± 28.28	(−)3.67 ± 20.93*
FVR, mmHg.100 mL/mL	8.33 ± 10.74	3.57 ± 10.30	1.59 ± 8.96*
FVR, %	23.49 ± 24.53	12.68 ± 30.38	4.32 ± 23.90*

SBP, systolic blood pressure; DBP, diastolic blood pressure; MAP, mean arterial pressure; HR, heart rate; FBF, forearm blood flow; FVR, forearm vascular resistance; bpm, beats per minute; Δ, delta; DGB, device-guided breathing.

*P < 0.05, **P < 0.001, tertile 3 versus tertile 1.

DGB significantly reduced blood pressure, with an effect that was similar across the tertiles of normetanephrine. DGB also reduced FBF but with an effect that was attenuated in the high compared with the low normetanephrine group: FBF reduction of −3.7 ± 20.9% in the highest compared with −18.6 ± 15.2% in the lowest tertiles of normetanephrine (*P* = 0.01). The reduction in heart rate by DGB was greater in the highest compared with the lowest tertile of normetanephrine.

**Fig. 2:**
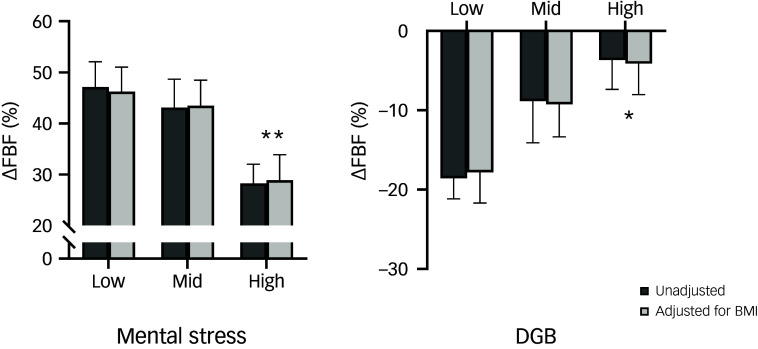
Forearm blood flow (FBF) responses to mental stress and device-guided breathing (DGB) stratified according to tertiles of plasma normetanephrine (NM). The values are unadjusted and adjusted for body mass index (BMI). *P < 0.05, **P < 0.001, tertile 3 (high NM) versus tertile 1 (low NM).

Similar results were seen for both mental stress and DGB when analysing changes in forearm vascular conductance to take into account concurrent changes in blood pressure (as expected, because these changes were similar across normetanephrine tertiles). Variation in FBF responses to mental stress and DGB across the tertiles of normetanephrine was similar whether adjusted or unadjusted for BMI (Fig. 2).

## Discussion

These data suggest a high prevalence of symptoms of depression and anxiety in individuals with hypertension, consistent with findings from other studies involving hypertensive populations.^3^ Specifically, in our sample of hypertensive individuals, 31–34% exhibited symptoms of moderate to severe depression based on PHQ-9 scores of ≥10, which is markedly higher than the 7–10% prevalence reported for the general adult population using the same assessment tool.^24,25^ A major finding of the present study is that symptoms of low mood and high anxiety are strongly associated with circulating plasma normetanephrine; thus, scores for PHQ-9 and QIDSSR-16 were approximately threefold higher for those in the highest compared with the lowest tertile of normetanephrine. These differences persisted following adjustment for BMI or sleep disturbance, factors that are associated with high sympathetic activity and potential mediators of the association.^26,27^

Higher plasma catecholamine concentrations and higher urinary catecholamine excretion have previously been reported in subjects with depression and anxiety, including in normotensive and untreated hypertensive populations.^12,13^ Hypertension is associated with increased levels of circulating catecholamines compared with those seen in normotensive subjects.^28^ However, in both depression and hypertension there is large range of concentrations of circulating catecholamines, with many individuals having concentrations that are within the range seen in the general population.^28–30^ The finding that depression and anxiety symptoms in those with hypertension are largely restricted to subjects with high concentrations of normetanephrine raises the possibility that these may reflect a common neurogenic mechanism linking these conditions. It is also possible that one condition may contribute directly to the onset or exacerbation of the other, underscoring the complex interplay between these comorbidities. However, one possibility is that chronic stress leads to persistent or more frequent/intense bursts of activation of the sympathetic nervous system via the ‘fight or flight’ reaction, which contributes to hypertension and can also result in anxiety and secondary depression.^31^ Chronic stress is known to increase activation of the sympathetic nervous system and is a risk factor for development of hypertension;^8^ it forms the basis of the most well-known animal model of depression and is frequently associated with depression in humans.^9^ Numerous mechanisms have been proposed to explain the link between chronic stress and depression, including effects secondary to alterations in immune function and inflammation induced by chronic activation of the hypothalamic–pituitary–adrenal axis.^9^ A direct effect is thought to involve activation of noradrenergic neurons in the locus coeruleus.^10^ The locus coeruleus is a centre implicated in attention and arousal, with projections throughout the brain, and modulates outflow from the midbrain to the spinal cord, regulating peripheral sympathetic nervous system activity.^11^ Norepinephrine released by these neurons engages with low-affinity α-adrenergic receptors in the prefrontal cortex (PFC), leading to a generalised reduction in neuronal activity and dendritic atrophy primarily in the dendritic spines of pyramidal neurons within the PFC, which is commonly observed in depression.^32,33^ It is also thought to decrease the level and function of the inhibitory neurotransmitter GABA, which is critical for maintaining the balance between excitatory and inhibitory signalling in cortical circuits. A deficit in GABAergic signalling is associated with increased cortical excitability and dysregulated neural networks involved in emotional regulation and stress response, both of which are central to the pathophysiology of depression.^34^

Seeking support for such a direct role of stress on the locus coeruleus and its projections, we examined vasomotor responses to mental stress and DGB. These are acute interventions that act on the same pathways as does chronic stress: PFC and noradrenergic neurons in the locus coeruleus.^32,35,36^ Mental stress activates a number of centres in the PFC,^37,38^ which then leads to peripheral vascular responses, the most notable of which is an increase in FBF.^39^ At a local level, forearm vasodilation is mediated by release of the neurotransmitter and vasodilator nitric oxide (NO) synthesised from neuronal NO synthase (nNOS), probably in skeletal muscle.^39,40^ Interconnections between the PFC and peripheral nNOS activation have not been fully characterised, but are likely to involve projections from the PFC via the locus coeruleus to the autonomic nervous system (ANS).^10^ Slowing the expiratory phase of breathing via DGB has long been known to reduce stress and alter the balance of sympathetic and parasympathetic activity in the ANS in favour of a reduction in sympathetic activity and increase in parasympathetic activity.^35^ It is now thought that alterations in breathing pattern are detected by specific neurons in the preBötzinger complex, the primary breathing rhythm generator.^41^ These neurons project onto and regulate noradrenergic neurons in the locus coeruleus, with slow breathing reducing activation of the latter,^41^ an effect opposite to mental stress. That subjects with hypertension and raised plasma normetanephrine concentrations had both mood disturbance and impaired vasomotor responses to mental stress and DGB is, therefore, consistent with involvement of the PFC and locus coeruleus in this hypertension/mood phenotype.

Both hypertension and affective disorders are highly heterogenous conditions with respect to aetiology, clinical characteristics and response to treatment. That the overlap of these two conditions may identify a specific phenotype is likely to have implications for the response to treatment, and raises the possibility that treatment of one condition could benefit the other. Early experiences with drugs such as hexamethonium, a ganglion-blocking agent for hypertension, did not suggest any benefit on depressive symptoms.^42^ However, it is possible that drugs acting in the central rather than peripheral nervous system to block sympathetic activity could improve depression. Identification of noradrenergic neurons in the projections of the locus coeruleus raises the possibility that α_1_ antagonists or α_2_ agonists that reduce central noradrenergic activity could benefit both conditions. There is some indirect evidence for this in that the α_1_ antagonist prazosin and α_2_ agonist clonidine are used in the treatment of post-traumatic stress disorder (PTSD) and may also improve depressive symptoms, although a recent large trial of prazosin for PTSD was negative.^43,44^ The atypical antipsychotic quetiapine is being increasingly used for depression,^45^ has α_1_ antagonist properties and reduces blood pressure, a feature shared by many other antipsychotics.^46^ In targeting an underlying disturbance of the hypertension/mood phenotype, such drugs may be particularly effective for their primary indication as well as treating the associated comorbidity. Conversely, noradrenergic uptake inhibitors that increase noradrenergic activity, such as duloxetine and venlafaxine, could be less effective in depression associated with hypertension and exacerbate the latter. However, we stress that this is purely speculative and needs to be tested in clinical trials.

### Limitations

Our study is subject to several limitations. We studied a relatively small sample of individuals referred to a secondary hypertension service, the majority of whom had well-controlled blood pressure and were on treatment with anti-hypertensive medications that could potentially have influenced normetanephrine levels. Further studies with a larger sample size, including in primary care populations, will be required to verify the present findings, in order to examine to what extent they may be influenced by treatment and common comorbidities such as obesity, obstructive sleep apnoea and diabetes. Additionally, participants did not undergo diagnostic interviews to confirm depression or anxiety, and symptom prevalence was based on questionnaire assessments which, although psychometrically robust with high test–retest reliability and strong validity, may not fully capture diagnostic criteria. The study also did not include a control group of a normotensive population, limiting direct comparisons. Although the association of affective disturbance and raised normetanephrine with impaired stress-mediated vasomotor responses is most readily explained by chronic stress impinging on pathways regulating central arousal and peripheral sympathetic nerve activity, we cannot make firm conclusions regarding causality from the results of the present study. Further studies examining the results of interventions that reduce chronic stress will be required to test this hypothesis.

In conclusion, a hyperadrenergic state in hypertension is associated with affective disturbance and impaired stress-modulated vasomotor responses. This association may be mediated by chronic stress impinging on pathways regulating central arousal and peripheral sympathetic nerve activity. The possibility that affective disturbance associated with hypertension may identify a phenotype with distinct neurophysiology should be explored.

## Data Availability

All data requests should be submitted to the corresponding author for consideration. Access to anonymised data may be granted following review.

## References

[1] Davidson K , Jonas BS , Dixon KE , Markovitz JH. Do depression symptoms predict early hypertension incidence in young adults in the CARDIA study? Coronary Artery Risk Development in Young Adults. Arch Intern Med 2000; 160: 1495–500. 10826464 10.1001/archinte.160.10.1495

[2] Rubio-Guerra AF , Rodriguez-Lopez L , Vargas-Ayala G , Huerta-Ramirez S , Serna DC , Lozano-Nuevo JJ. Depression increases the risk for uncontrolled hypertension. Exp Clin Cardiol 2013; 18: 10–2. 24294029 PMC3716493

[3] Li Z , Li Y , Chen L , Chen P , Hu Y. Prevalence of depression in patients with hypertension: a systematic review and meta-analysis. Medicine 2015; 94: e1317. 26252317 10.1097/MD.0000000000001317 PMC4616591

[4] Meng L , Chen D , Yang Y , Zheng Y , Hui R. Depression increases the risk of hypertension incidence: a meta-analysis of prospective cohort studies. J Hypertens 2012; 30: 842–51. 22343537 10.1097/HJH.0b013e32835080b7

[5] Maatouk I , Herzog W , Böhlen F , Quinzler R , Löwe B , Saum KU , et al. Association of hypertension with depression and generalized anxiety symptoms in a large population-based sample of older adults. J Hypertens 2016; 34: 1711–20. 27341438 10.1097/HJH.0000000000001006

[6] Hare DL , Toukhsati SR , Johansson P , Jaarsma T. Depression and cardiovascular disease: a clinical review. Eur Heart J 2014; 35: 1365–72. 24282187 10.1093/eurheartj/eht462

[7] Fuchs FD , Whelton PK. High blood pressure and cardiovascular disease. Hypertension 2020; 75: 285–92. 31865786 10.1161/HYPERTENSIONAHA.119.14240 PMC10243231

[8] Spruill TM. Chronic psychosocial stress and hypertension. Curr Hypertens Rep 2010; 12: 10–6. 20425153 10.1007/s11906-009-0084-8 PMC3694268

[9] Yang L , Zhao Y , Wang Y , Liu L , Zhang X , Li B , et al. The effects of psychological stress on depression. Curr Neuropharmacol 2015; 13: 494–504. 26412069 10.2174/1570159X1304150831150507 PMC4790405

[10] Morris LS , McCall JG , Charney DS , Murrough JW. The role of the locus coeruleus in the generation of pathological anxiety. Brain Neurosci Adv 2020; 4: 2398212820930321. 32954002 10.1177/2398212820930321 PMC7479871

[11] Samuels ER , Szabadi E. Functional neuroanatomy of the noradrenergic locus coeruleus: its roles in the regulation of arousal and autonomic function part II: physiological and pharmacological manipulations and pathological alterations of locus coeruleus activity in humans. Curr Neuropharmacol 2008; 6: 254–85. 19506724 10.2174/157015908785777193 PMC2687931

[12] Hughes JW , Watkins L , Blumenthal JA , Kuhn C , Sherwood A. Depression and anxiety symptoms are related to increased 24-hour urinary norepinephrine excretion among healthy middle-aged women. J Psychosom Res 2004; 57: 353–8. 15518669 10.1016/j.jpsychores.2004.02.016

[13] Paine NJ , Watkins LL , Blumenthal JA , Kuhn CM , Sherwood A. Associations of depressive and anxiety symptoms with 24-hour urinary catecholamines in individuals with untreated high blood pressure. Psychosom Med 2015; 77: 136–44. 25647750 10.1097/PSY.0000000000000144 PMC5119914

[14] Peaston RT , Weinkove C. Measurement of catecholamines and their metabolites. Ann Clin Biochem 2004; 41: 17–38. 14713382 10.1258/000456304322664663

[15] Williams B , Mancia G , Spiering W , Rosei EA , Azizi M , Burnier M , et al. ESC/ESH 354 Guidelines for the management of arterial hypertension: The Task Force for the management of arterial hypertension of the European Society of Cardiology and the European Society of Hypertension. J Hypertens 2018; 36: 1953–2041. 30234752 10.1097/HJH.0000000000001940

[16] Trivedi MH , Rush AJ , Ibrahim HM , Carmody TJ , Biggs MM , Suppes T , et al. The Inventory of Depressive Symptomatology, clinician rating (IDS-C) and self-report (IDS-SR), and the Quick Inventory Depressive Symptomatology, clinician rating (QIDS-C) and self-report (QIDS-SR) in public sector patients with mood disorders: a psychome. Psychol Med 2004; 34: 73–82. 14971628 10.1017/s0033291703001107

[17] Kroenke K , Spitzer RL , Williams JBW. The PHQ-9: validity of a brief depression severity measure. J Gen Intern Med 2001; 16: 606–13. 11556941 10.1046/j.1525-1497.2001.016009606.x PMC1495268

[18] Löwe B , Decker O , Müller S , Brähler E , Schellberg D , Herzog W , et al. Validation and standardization of the generalized anxiety disorder screener (GAD-7) in the general population. Med Care 2008; 46: 266–74. 18388841 10.1097/MLR.0b013e318160d093

[19] Nagappa M , Liao P , Wong J , Auckley D , Ramachandran SK , Memtsoudis S , et al. Validation of the stop-bang questionnaire as a screening tool for obstructive sleep apnea among different populations: a systematic review and meta-analysis. PLoS One 2015; 10: e0143697. 26658438 10.1371/journal.pone.0143697 PMC4678295

[20] Johns MW. Reliability and factor analysis of the Epworth Sleepiness Scale. Sleep 1992; 15: 376–81. 1519015 10.1093/sleep/15.4.376

[21] MacLeod CM. Half a century of research on the stroop effect: an integrative review. Psychol Bull 1991; 109: 163–203. 2034749 10.1037/0033-2909.109.2.163

[22] Parris BA , Wadsley MG , Hasshim N , Benattayallah A , Augustinova M , Ferrand L. An fMRI study of response and semantic conflict in the Stroop task. Front Psychol 2019; 10: 2426. 31736827 10.3389/fpsyg.2019.02426 PMC6834775

[23] Cohen J. Statistical Power Analysis for the Behavioral Sciences 2nd ed. Lawrence Erlbaum Associates, 1988.

[24] Thibodeau MA , Asmundson GJG. The PHQ-9 assesses depression similarly in men and women from the general population. Pers Individ Dif 2014; 56: 149–53.

[25] Hinz A , Mehnert A , Kocalevent RD , Brähler E , Forkmann T , Singer S , et al. Assessment of depression severity with the PHQ-9 in cancer patients and in the general population. BMC Psychiatry 2016; 16: 22. 26831145 10.1186/s12888-016-0728-6 PMC4736493

[26] Esler M , Straznicky N , Eikelis N , Masuo K , Lambert G , Lambert E. Mechanisms of sympathetic activation in obesity-related hypertension. Hypertension 2006; 48: 787–96. 17000932 10.1161/01.HYP.0000242642.42177.49

[27] Narkiewicz K , Somers VK. Sympathetic nerve activity in obstructive sleep apnoea. Acta Physiol Scand 2003; 177: 385–90. 12609010 10.1046/j.1365-201X.2003.01091.x

[28] Goldstein DS. Plasma norepinephrine in essential hypertension. A study of the studies. Hypertension 1981; 3: 48–52. 7203605 10.1161/01.hyp.3.1.48

[29] Roy A , Pickar D , Linnoila M , Potter WZ. Plasma norepinephrine level in affective disorders: relationship to melancholia. Arch Gen Psychiatry 1985; 42: 1181–5. 4074110 10.1001/archpsyc.1985.01790350055010

[30] Wyatt RJ , Portnoy B , Kupfer DJ , Snyder F , Engelman K. Resting plasma catecholamine concentrations in patients with depression and anxiety. Arch Gen Psychiatry 1971; 24: 65–70. 5538854 10.1001/archpsyc.1971.01750070067009

[31] Holwerda SW , Luehrs RE , Gremaud AL , Wooldridge NA , Stroud AK , Fiedorowicz JG , et al. Relative burst amplitude of muscle sympathetic nerve activity is an indicator of altered sympathetic outflow in chronic anxiety. J Neurophysiol 2018; 120: 11–22. 29537916 10.1152/jn.00064.2018 PMC6093954

[32] Arnsten AFT , Raskind MA , Taylor FB , Connor DF. The effects of stress exposure on prefrontal cortex: translating basic research into successful treatments for post-traumatic stress disorder. Neurobiol Stress 2015; 1: 89–99. 25436222 10.1016/j.ynstr.2014.10.002 PMC4244027

[33] Qiao H , Li M-X , Xu C , Chen H-B , An S-C , Ma X-M. Dendritic spines in depression: what we learned from animal models. Neural Plast 2016; 2016: 8056370. 26881133 10.1155/2016/8056370 PMC4736982

[34] Luscher B , Shen Q , Sahir N. The GABAergic deficit hypothesis of major depressive disorder. Mol Psychiatry 2011; 16: 383–406. 21079608 10.1038/mp.2010.120 PMC3412149

[35] Zaccaro A , Piarulli A , Laurino M , Garbella E , Menicucci D , Neri B , et al. How breath-control can change your life: a systematic review on psycho-physiological correlates of slow breathing. Front Hum Neurosci 2018; 12: 353. 30245619 10.3389/fnhum.2018.00353 PMC6137615

[36] Yennu A , Tian F , Smith-Osborne A , Gatchel RJ , Woon FL , Liu H. Prefrontal responses to Stroop tasks in subjects with post-traumatic stress disorder assessed by functional near infrared spectroscopy. Sci Rep 2016; 6: 30157. 27452397 10.1038/srep30157 PMC4995363

[37] Milham MP , Banich MT , Barad V. Competition for priority in processing increases prefrontal cortex’s involvement in top-down control: an event-related fMRI study of the Stroop task. Cogn Brain Res 2003; 17: 212–22. 10.1016/s0926-6410(03)00108-3 12880892

[38] Van Veen V , Carter CS. Separating semantic conflict and response conflict in the Stroop task: a functional MRI study. Neuroimage 2005; 27: 497–504. 15964208 10.1016/j.neuroimage.2005.04.042

[39] Dietz NM , Rivera JM , Eggener SE , Fix RT , Warner DO , Joyner MJ. Nitric oxide contributes to the rise in forearm blood flow during mental stress in humans. J Physiol 1994; 480: 361–8. 7869251 10.1113/jphysiol.1994.sp020366 PMC1155852

[40] Seddon MD , Chowienczyk PJ , Brett SE , Casadei B , Shah AM. Neuronal nitric oxide synthase regulates basal microvascular tone in humans in vivo. Circulation 2008; 117: 1991–6. 18391107 10.1161/CIRCULATIONAHA.107.744540

[41] Yackle K , Schwarz LA , Kam K , Sorokin JM , Huguenard JR , Feldman JL , et al. Breathing control center neurons that promote arousal in mice. Science 2017; 355: 1411–5. 28360327 10.1126/science.aai7984 PMC5505554

[42] Paton WDM. Paralysis of autonomic ganglia and the therapeutic effects of ganglion-blocking drugs. Br Med J 1951; 1: 773–8. 14821528 10.1136/bmj.1.4710.773 PMC2068726

[43] Wendell KR , Maxwell ML. Evaluation of clonidine and prazosin for the treatment of nighttime posttraumatic stress disorder symptoms. Fed Pract 2015; 32: 8–14. PMC6364812 30766029

[44] Raskind MA , Peskind ER , Chow B , Harris C , Davis-Karim A , Holmes HA , et al. Trial of prazosin for post-traumatic stress disorder in military veterans. New Engl J Med 2018; 378: 507–17. 29414272 10.1056/NEJMoa1507598

[45] Daly EJ , Trivedi MH. A review of quetiapine in combination with antidepressant therapy in patients with depression. Neuropsychiatr Dis Treat 2007; 3: 855–67. 19300621 10.2147/ndt.s1862 PMC2656328

[46] Tan HH , Hoppe J , Heard K. A systematic review of cardiovascular effects after atypical antipsychotic medication overdose. Am J Emerg Med 2009; 27: 607–16. 19497468 10.1016/j.ajem.2008.04.020 PMC2759317

